# Oxygen reserve index for non-invasive early hypoxemia detection during endotracheal intubation in intensive care: the prospective observational NESOI study

**DOI:** 10.1186/s13613-021-00903-8

**Published:** 2021-07-17

**Authors:** Hugo Hille, Aurelie Le Thuaut, Emmanuel Canet, Jeremie Lemarie, Laura Crosby, Gregoire Ottavy, Charlotte Garret, Maelle Martin, Amelie Seguin, Pauline Lamouche-Wilquin, Jean Morin, Olivier Zambon, Arnaud-Felix Miaihle, Jean Reignier, Jean-Baptiste Lascarrou

**Affiliations:** 1 Médecine Intensive Réanimation, Centre Hospitalier Universitaire de Nantes, Nantes, France; 2 Plateforme de Méthodologie Et Biostatistique, Direction de La Recherche de L’Innovation, Centre Hospitalier Universitaire de Nantes, Nantes, France; 3 Paris Cardiovascular Research Centre, Université de Paris, INSERM, Paris, France; 4 Service de Médecine Intensive Réanimation, Centre Hospitalier Universitaire Hôtel-Dieu, 30 Bd. Jean Monnet, 44093 Nantes Cedex 1, France

**Keywords:** Intensive care, Intubation, Oxygenation, Monitoring

## Abstract

**Background:**

To evaluate the ability of the oxygen reserve index (ORI) to predict the occurrence of mild hypoxemia (defined as SpO_2_  <  97%) during endotracheal intubation (ETI) of patients in the intensive care unit (ICU).

**Methods:**

This observational single-centre study included patients without hypoxemia (defined as SpO_2_/FiO_2_  >  214) who required ETI in the ICU. Patients were followed during preoxygenation and ETI then until hospital discharge and/or day 28. We recorded cases of mild hypoxemia, moderate (SpO_2_  <  90%) and severe (SpO_2_  <  80%) hypoxemia, moderate arterial hypotension (systolic arterial pressure  <  90 mmHg), oesophageal intubation, aspiration, cardiac arrest, and death.

**Results:**

Between January 2019 and July 2020, 56 patients were included prospectively and 51 patients were analysed. Twenty patients had mild hypoxemia between the end of preoxygenation and the end of intubation; in 10 of these patients, the decrease in SpO_2_ below 97% was preceded by an ORI  <  0.4, the median time difference being 81 s [interquartile range, 34–146]. By multivariable analysis, a higher ORI (by 0.1 increase) value during preoxygenation was associated with absence of hypoxemia (odds ratio, 0.76; 95% confidence interval, 0.61;0.95; *P * =  0.0141).

**Conclusion:**

In non-hypoxemic patients, the 81-s [34–146] median time between the ORI decrease below 0.4 and the SpO_2_ decrease below 97% during apnoea may allow preventive action. A higher ORI value during preoxygenation was independently protective against hypoxemia. Whether these findings also apply to hypoxemic patients, and the clinical impact of a preoxygenation strategy based on ORI monitoring, remain to be evaluated prospectively.

*Trial Registration* ClinicalTrial.gov, #NCT03600181.

**Supplementary Information:**

The online version contains supplementary material available at 10.1186/s13613-021-00903-8.

## Introduction

Endotracheal intubation (ETI) is performed in many patients admitted to the intensive care unit (ICU), the proportion being 22% in a multicenter study [1]. The circumstances that surround ETI in the ICU are associated with a high complication rate of up to 50% [1–4]. The serious complications are severe hypoxemia (26%), severe hypotension (25%), cardiac arrest (1–3%), and death (0.5–3%) [1, 4, 5]. Severe hypoxemia, which can be fatal [7], is more common when the patient is hypoxemic before intubation and/or intubation is difficult [8, 9].

Predicting the occurrence of hypoxemia during ETI is challenging [10]. Preoxygenation reduces the risk by prolonging the safe apnoea time and is therefore universally recommended [11, 12]. Preoxygenation consists in giving pure oxygen to wash out the nitrogen contained in the lungs at functional residual capacity. The effectiveness of preoxygenation is evaluated experimentally and in the operating room by the expired fraction of oxygen (FeO_2_), but this marker has limitations in the emergency setting (sensitivity to leaks) and is not available in the ICU [13]. Recent studies have evaluated various preoxygenation devices with contradictory results [6, 14–16], and currently no device ensures that desaturation will not occur during intubation [17]. Pulsed oxygen saturation (SpO_2_) measured by pulse oximetry—which is the parameter monitored in intensive care—only detects hypoxemia at a late stage. Thus, at present, the effectiveness of preoxygenation cannot be evaluated in intensive care. The absence and/or low quality of preoxygenation was associated with the occurrence of cardiac arrest during the peri-intubation period in a retrospective analysis of a multicentre prospective database that had 1847 ETI procedures [7]. Thus, optimising preoxygenation is a crucial goal. To optimise preoxygenation, the availability of a reliable predictor of desaturation would be valuable.

The oxygen reserve index (ORI) is a new oxygenation monitoring parameter measured continuously and noninvasively by a specific pulse oximeter device manufactured by Masimo (Irvine, CA). It provides a dimensionless index from 0.0 (PaO_2_  ≥  100 mm Hg) to 1.0 (PaO_2_  ≥  200 mm O_2_). Thus, the ORI supplies information beyond the range explored by SpO_2_.

The objective of this prospective observational pilot study was to evaluate the potential role for ORI in providing early warning that hypoxemia will occur during ETI of patients in the ICU.

## Patients and methods

### Study design

We conducted a prospective observational proof-of-concept single-centre study. We collected the data in the medical ICU of the Nantes University Hospital (Nantes, France), which has 25 ICU beds and five intermediate-care beds.

The study was approved by our ethics committee on September, 2018 (CPP Ile de France 1, ID-RCB: 2018-A01288-47) and was open to inclusion on ClinicalTrials.gov on January 30, 2019 (#NCT03600181). Written informed consent to participation was obtained from each patient or proxy. According to French legislation, patients who were not competent and had no proxy available were included if they met the selection criteria then asked for their consent as soon as they recovered competency.

### Oxygen reserve index

The oxygen reserve index (ORI) is a nondimensional index that ranges from 1 (high reserve) to 0 (no reserve) and is measured by optically detecting changes in mixed venous oxygen saturation (SvO_2_) after oxygen saturation (SaO_2_) reaches 100%. It is measured by a multi-wavelength pulse co-oximeter placed on the tip of a finger (Rainbow SET, Masimo) [18]. The device analyses variations in the pulsatile blood absorption of incident light at both the arterial and venous levels. It is a relative indicator of PaO_2_ changes in the moderate hyperoxemia range. When pure oxygen is administered, SaO_2_ reaches 100% when PaO_2_ reaches 100 mmHg. Beyond that, PaO_2_ continues to increase, both SaO_2_ and SpO_2_ remain at 100%, and the ORI increases non-linearly from 0.00 (PaO_2_–100 mm Hg) to 1.00 (PaO_2_–200 mmHg).

### Patient selection

Inclusion criteria were ICU admission with a need for ETI and a SpO_2_/FiO_2_ ratio above 214. The SpO_2_/FiO_2_ ratio was measured during non-invasive ventilation (NIV) or high-flow oxygen therapy. For conventional oxygen therapy, the fraction of inspired oxygen (FiO_2_) was calculated as follows: FiO_2_ =  0.21  +  O_2_ flow·0.03 [19].

Exclusion criteria were age younger than 18 years, indication to use an alternate tool to perform ETI (unstable spinal cord injury for example), insufficient time to include the patient (e.g., cardiac arrest), pregnancy or breastfeeding, being a correctional services inmate, being under guardianship, or not being covered by the French statutory health insurance system.

Patients were secondarily excluded in the event of an ORI device malfunction, failure of ORI recording by the computer, ORI remaining constant at 1 throughout preoxygenation, and SpO_2_  <  97% throughout preoxygenation [20].

### Data collection

Standardised forms were used to record the following data: patient’s baseline characteristics [including difficult intubation criteria: Mallampati score, thyromental distance  <  65 mm, mouth opening  <  35 mm, limited cervical mobility, sleep apnoea, body mass index (BMI)  >  35], main reason for ETI (neurological, respiratory, cardiovascular, or other), use of a bougie and/or other devices (e.g., laryngeal mask airway or videolaryngoscope), duration of preoxygenation, total ETI duration (from anaesthesia induction to capnography over more than three cycles indicating proper endotracheal tube position), and baseline SpO_2_. We also collected SpO_2_ and ORI at four time points: beginning of preoxygenation, end of preoxygenation, during ETI and just after successful ETI. SpO_2_ drops below 97%, 90%, and 80% between anaesthesia induction and successful ETI were recorded. Complications were recorded as death, cardiac arrest, and systolic blood pressure drop to less than 90 mmHg [21]. Case-report forms were completed in real time by a dedicated clinical research nurse. Each case report form was then introduced into an electronic file (Excel, Microsoft Corporation, Redmond, WA).

### Outcomes

The primary endpoint was the time between the ORI decrease below 0.4 and the SpO_2_ decrease below 97% during ETI (between the end of preoxygenation and confirmation that the tube was in the trachea). The value of 0.4 for ORI was chosen in agreement with the only available study [22].

The secondary objective of our study was to determine whether a decline in ORI during preoxygenation predicted the occurrence of SpO_2_  <  97% during ETI.

### Intubation procedure

Once the decision to perform ETI was made and consent obtained, an ORI device (Rainbow^®^ Lite SET-1 Adt sensors, Revision M, Masimo) was applied to the patient’s 3rd or 4th fingertip on the contralateral side of the non-invasive blood pressure monitoring device. The sensor was covered to protect it from light. SpO_2_ and ORI values displayed on the Rad7^®^ monitor were recorded every 2 s and transferred to a laptop computer throughout the ETI procedure. The patient’s physicians were not aware of the ORI values.

ETI was performed according to the standardised protocol used in the ICU of the Nantes University Hospital [21]. The choice of the preoxygenation device, anaesthesia induction agents, and ETI device was at the discretion of the physician in charge. The recommended preoxygenation duration was 3 min.

The procedure began with the initiation of preoxygenation and ended with confirmation that the tube was in the trachea. The end of preoxygenation was defined as the induction of anaesthesia. The duration of ETI was the time from the end of preoxygenation and confirmation by capnography of correct tube position. Neither apneic oxygenation nor apneic ventilation [23] was part of our local protocol during management of the study patients.

### Sample size

In the absence of critical care data, we estimated the time to an SpO_2_ decrease to 97% at 60  ±  30 s [22, 24] and we assumed that the ORI would decrease below 0.4 (warning cut-off determined by the manufacturer and consistent with data from a paediatric study [22]), 30 s before SpO_2_ decreased below 97%. We estimated the median time between these two decreases, with the 95% confidence interval (95% CI) and an accuracy of 10 s. Assuming a standard deviation of 30 s, 35 patients were needed [25]. According to Szmuk et al. [22], it was expected that 28% of patients would have a secondary exclusion criterion, and we therefore decided to include 50 patients.

### Statistical analysis

Qualitative data were described as frequency and percentage and quantitative data as mean  ±  SD and median [interquartile range].

The median time between the ORI decrease below 0.4 and the SpO_2_ decrease below 97% during ETI was estimated with its 95% confidence interval (95% CI).

The performance of the ORI for predicting SpO_2_  <  97% during ETI was estimated by the area under the ROC curve with its 95% CI. To evaluate the informative value of the ORI signal, a multivariate logistic regression model with the occurrence of SpO_2_  <  97% as a predictor variable was constructed. The variables included by step-down selection were the ORI and the variables selected a priori as clinically relevant [10] (age, gender, BMI, and ETI duration).

All tests were two-tailed with a significance level of 0.05. No imputation strategy was used. The statistical analyses were done using Stata^®^ statistical software (version 13; StataCorp LP, College Station, TX).

## Results

### Patients

Between February 2019 and July 2020, 56 patients were included. Main reasons for non-inclusion were SpO_2_/FiO_2_  <  214 (*n*  =  114, 76%) followed by omission (*n*  =  21, 14%) (Additional file 1: Figure S1). Among the 56 included patients, 5 were secondarily excluded, leaving 51 for in the analysis. Table 1 reports their main features. No patient had a history of difficult intubation, the Mallampati score was 1/4 in 36 (70.6%) patients, and 22 (43%) patients met none of the criteria for difficult ETI.

**Table 1:** Patients’ baseline characteristics

Age, years	59 [43–66]
Gender, female	22 (43.14%)
Body mass index, kg m^−2^	24.22 [20.81–29.73]
Past medical history	
Active smoking	13 (25.49%)
Chronic arterial hypertension	12 (23.52%)
Diabetes mellitus	7 (13.72%)
Cancer and/or immunodepression	9 (17.64%)
Cirrhosis	9 (17.64%)
Chronic obstructive apnoea	4 (7.84%)
History of hepatic encephalopathy	3 (5.88%)
Chronic respiratory disease	2 (3.92%)
Neuromuscular disease	2 (3.92%)
Other	9 (17.64%)
Reason for ICU admission	
Acute neurologic failure	29 (56.80%)
Acute respiratory failure	10 (19.61%)
Shock	7 (13.73%)
Acute renal failure	5 (9.80%)
Reason for endotracheal intubation	
Drug and/or alcohol poisoning	9 (17.65%)
Hepatic encephalopathy	5 (9.80%)
Post-extubation respiratory distress	5 (9.80%)
Acute respiratory failure	4 (7.84%)
Septic shock	4 (7.84%)
Epileptic condition	3 (5.88%)
Guillain–Barré syndrome	3 (5.88%)
Myasthenia gravis	2 (3.92%)
Encephalitis	2 (3.92%)
Haemorrhagic shock	2 (3.92%)
Acute pancreatitis	2 (3.92%)
Delirium tremens	1 (1.96%)
Meningitis	1 (1.96%)
Inhalation pneumopathy	1 (1.96%)
Haemorrhagic stroke	1 (1.96%)
Other	6 (11.76%)
SAPS II	46 [30–62]
SOFA score at ICU admission	6 [3–10]
Arterial blood gas values at ICU admission (*n* = 30)	
pH	7.39 [7.27–7.45]
PaCO_2_, mmHg	37.13 [28.50–43.50]
PaO_2_, mmHg	101.38 [76–145.50]
PaO_2_/FiO_2_	323 [233–479]
SaO_2_, %	97.75 [95.25–99.15]
CO_2_, mmol/L	24.50 [17.40–27.40]
SpO_2_/FiO_2_ at ICU admission	388 [278–457]
Arterial lactate, mmol L^−1^	2.10 [1.50–4.60]

The data are median [25th–75th percentiles] or number (percentage)

*ICU* intensive care unit; *SAPSII* Simplified Acute Physiology Score version II; *SOFA score* Sequential Organ Failure Assessment score

### Procedure

Preoxygenation was performed with a bag valve mask (*n*  =  40, 78.4%) or NIV (*n*  =  11, 21.6%) and ETI with a standard laryngoscope (*n*  =  48, 94.1%) or a videolaryngoscope (*n*  =  3, 5.9%). A bougie was used in 16 (31.4%) patients. The anaesthetic agents used for induction were etomidate in 43 (84.3%) patients and propofol in 8 patients (15.7%), for hypnosis; for neuromuscular blockade, succinylcholine was used in 41 (80.4%) patients and rocuronium in 9 (17.6%) patients, with the remaining patient receiving no neuromuscular blocker. The operator was a resident for 48 (94.1%) patients; residents were closely supervised by senior intensivists.

ETI was achieved in all patients, with a median number of attempts of 1 [1, 2] and a proportion of successful first attempts of 74.5% (*n*  =  38). In the remaining 13 patients, ETI was achieved after at least one additional attempt; the reasons for first-attempt failure were poor glottis visibility in 5 (38.5%) patients and tracheal catheterisation failure in 8 (61.5%) patients. ETI was difficult in 3 (5.9%) patients (i.e., required at least three attempts). The only patient who needed more than three attempts was intubated on the seventh attempt. At the time of exposure, head elevation was required in 7 (13.7%) patients, backwards-upwards-rightwards pressure (BURP) on the larynx in 14 (27.5%) patients, and the Sellick manoeuvre in 3 (5.9%) patients. The Cormack–Lehane grade was 1 or 2 in 45 (88.2%) patients and 3 or 4 in 6 (11.8%) patients. The median percentage of glottic opening seen (POGO) was 80 [70–100]. In 6 (11.8%) patients, face-mask ventilation was required after the first attempt due either to desaturation (*n*  =  3) or to first-attempt failure (*n*  =  3). The median lowest SpO_2_ during intubation was 98% [91–99] in patients who did not need face-mask ventilation and 72% [55–82] in those who did require face-mask ventilation before ETI. SpO_2_ fell below 97% in 20 patients.

ETI-related complications occurred in 24 (24/51, 47.1%) patients. By decreasing order of frequency, they consisted of arterial hypotension (*n*  =  18, 75%), moderate hypoxemia (SpO_2_  <  90%, *n * =  9, 37.5%), severe hypoxemia (SpO_2_  <  80%, *n*  =  4, 16.7%), oesophageal intubation (*n*  =  2, 8.3%), and aspiration (*n*  =  1, 4.1%). No patient had cardiorespiratory arrest or death due to ETI.

Overall, 9 (17.6%) patients died in the ICU. On day 28, 42 (82.4%) patients were alive. The median ICU length of stay was 5.36 days [2.9–15.8].

### Primary outcome

Of the 51 patients, 20 experienced an SpO_2_ fall below 97% between the end of preoxygenation and successful ETI. An ORI fall below 0.4 preceded this SpO_2_ fall under 97% in 10 of these patients. In the other 10 patients, when the SpO_2_ fall below 97% occurred, the ORI had not increased above 0.4. In the 10 patients with a warning ORI fall below 0.4 before the SpO_2_ fall below 97%, the median time between these two events was 81 s [34–146] and the median ETI duration was 303 s [180–648].

### Secondary outcomes

**Fig. 1:**
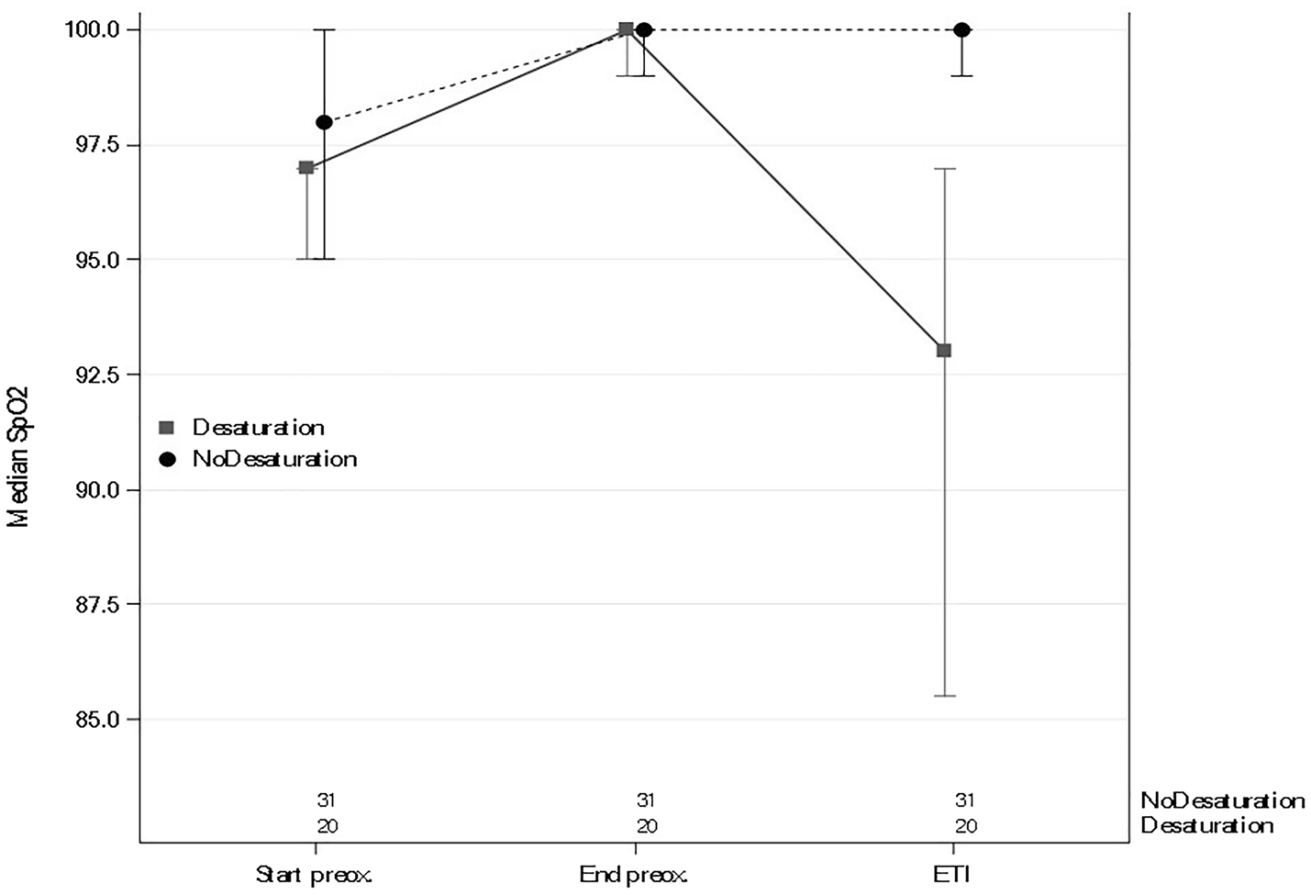
SpO_2_ changes during preoxygenation and endotracheal intubation (ETI)

**Fig. 2:**
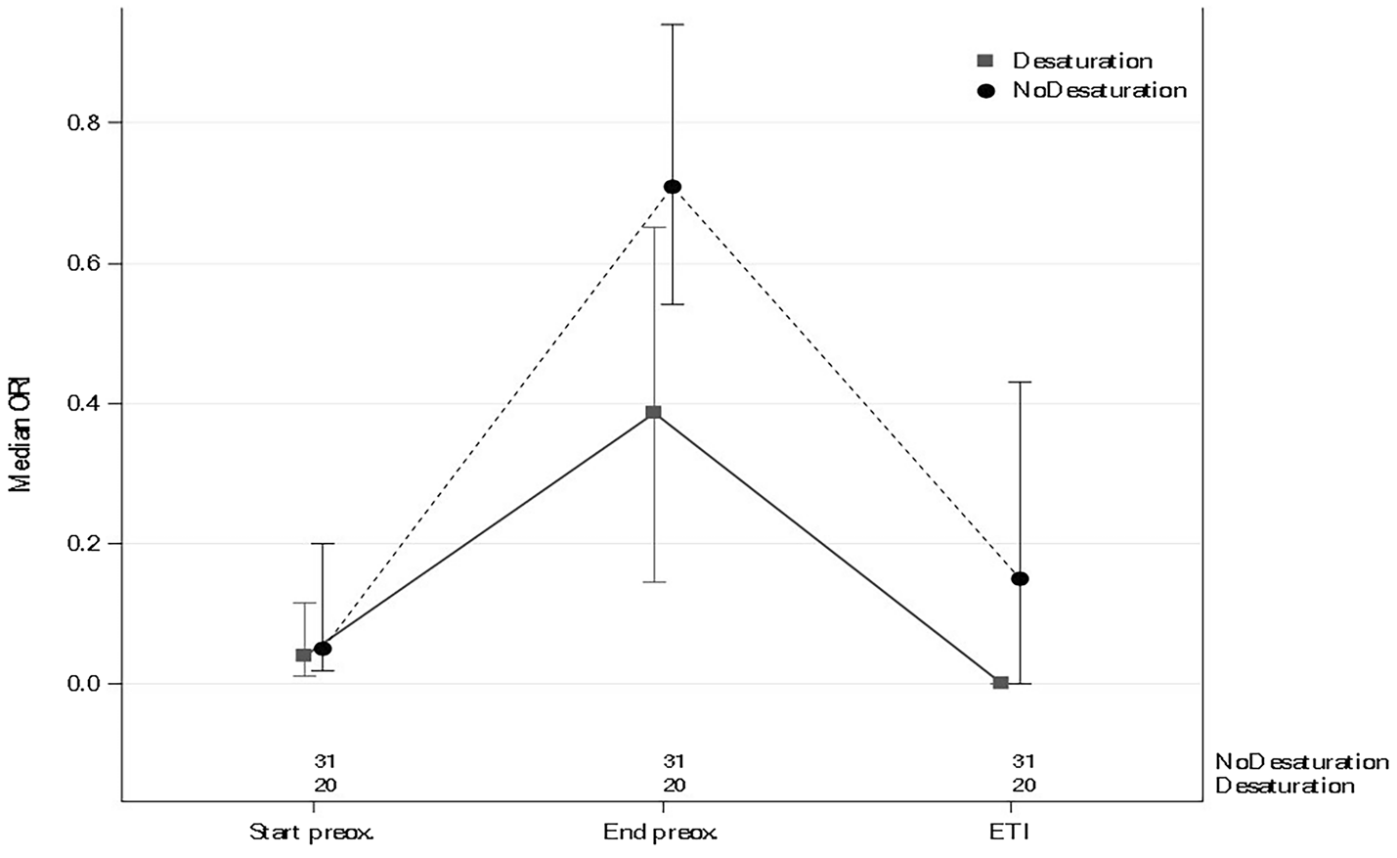
Changes in oxygen reserve index (ORI) in patients with and without hypoxemia during endotracheal intubation (ETI)

As noted above, of the 51 patients, 20 (39.2%) experienced SpO_2_  <  97% during intubation. Table 2 and Figs. 1 and 2 show the changes in SpO_2_ and ORI from the start of preoxygenation to the end of intubation. Additional file 2: Figure S2 shows changes in SpO_2_ and ORI in the subset of patients who had moderate hypoxemia and Additional file 3: Figure S3 shows changes in all patients.

**Table 2:** Comparison of peripheral oxygen saturation (SpO_2_) and oxygen reserve index (ORI) in patients with and without SpO_2_  <  97% during endotracheal intubation (ETI)

	Total *n* = 51	SpO_2_ ≥ 97% *n* = 31	SpO_2_ < 97% *n * = 20	*P* value
SpO_2_ at the start of preoxygenation, median [IQR]	97.00 [95.00–99.00]	98.00 [95.00–100.00]	97.00 [95.00–97.00]	0.0951
SpO_2_ at the end of preoxygenation, median [IQR]	100.00 [99.00–100.00]	100.00 [99.00–100.00]	100.00 [99.00–100.00]	0.5931
SpO_2_ at ETI, median [IQR]	99.00 [95.00–100.00]	100.00 [99.00–100.00]	93.00 [85.50–97.00]	< 0.0001
ORI at the start of preoxygenation, median [IQR]	0.04 [0.01–0.13]	0.05 [0.02–0.20]	0.04 [0.01–0.12]	0.2891
ORI at the end of preoxygenation, median [IQR]	0.62 [0.26–0.83]	0.71 [0.54–0.94]	0.39 [0.15–0.65]	0.0082
Change in ORI from start to end of preoxygenation, median [IQR]	0.46 [0.15–0.64]	0.52 [0.23–0.82]	0.17 [0.10–0.50]	0.0191
Highest ORI during preoxygenation, median [IQR]	0.67 [0.31–0.94]	0.77 [0.59–1.00]	0.49 [0.22–0.77]	0.0212
Changes in SpO_2_ from start to end of preoxygenation, median [IQR]	3.00 [1.00–4.00]	2.00 [0.00–4.00]	3.00 [2.00–5.00]	0.1181

*SpO*_*2*_ oxygen saturation by pulse oximeter; *IQR* interquartile range; *ETI* endotracheal intubation; *ORI* oxygen reserve index

The areas under the ROC curve for ORI during preoxygenation and at the end of preoxygenation predicted the occurrence of SpO_2_  <  97% during intubation (0.73; 95% CI 0.58–0.88 and 0.70; 95% CI 0.54–0.85, respectively). In contrast, the area under the ROC curve of SpO_2_ at the end of preoxygenation was poorly predictive of SpO_2_  <  97% during intubation (0.54; 95% CI 0.40–0.67).

By univariate analysis, longer ETI duration was associated with the occurrence of SpO_2_  <  97% during intubation (odds ratio, 1.00; 95% CI 1.00–1.01; *P * =  0.0474), whereas a higher ORI value during preoxygenation was associated with a less frequent occurrence of SpO_2_  <  97% (odds ratio, 0.09; 95% CI 0.01–0.69; *P * =  0.0199). The highest ORI value during preoxygenation remained significantly associated with a lower risk of SpO_2_  <  97% during ETI after adjustment for ETI duration and BMI (odds ratio, 0.76; 95% CI 0.61–0.95; *P * =  0.0141; Table 3).

**Table 3:** Multivariable analysis: factors associated with SpO_2_  <  97% during endotracheal intubation (ETI)

Variable	OR	95% CI	*P* value
Duration of ETI (seconds)	1.00	[1.00; 1.01]	0.0494
BMI > 25 kg·m^−2^	1.73	[0.45; 6.65]	0.1295
Highest ORI during preoxygenation	0.76	[0.61; 0.95]	0.0141

*OR* odds ratio; *95% CI* 95% confidence interval; *ETI* endotracheal intubation; *BMI* body mass index; *ORI* oxygen reserve index

## Discussion

The main findings from our study are that in critically ill patients who require ETI, are not hypoxemic, and whose ORI is above 0.4 during preoxygenation, the median time between the ORI decrease below 0.4 and the SpO_2_ decrease below 97% was 81 s [34–146] and that, by multivariate analysis, a higher ORI value during preoxygenation was associated with a lower risk of desaturation below 97% during ETI, after adjustment on ETI duration. The median highest ORI value during preoxygenation was higher in the group without desaturation below 97%. In contrast, no differences between the two groups were found for the SpO_2_ values at the beginning or end of preoxygenation or for the change in SpO_2_ between the beginning and end of preoxygenation.

Our results are consistent with those found in patients undergoing elective surgery, which were prospective observational studies with small sample sizes. Reported time intervals between the ORI decline and desaturation were 31.5 [19.0–34.3] s [22], 32.5 [18.8–51.3] s [20], and 48.4 [40.4–62.0] s [22]. Thus, the warning time in patients without critical illness was shorter than in our cohort. Possible explanations are the variations in cut-offs chosen to define hypoxemia, inclusion of children in some studies, use of the latest sensor model (revision M of revision L) in our study, possible continuation of oxygenation during the apneic period in previous studies, and/or successful ETI in some patients after a failed first attempt but before the occurrence of decreases in ORI and SpO_2_.

SpO_2_ changes during preoxygenation serve as a proxy for PaO_2_ changes, but neither evaluate the oxygen reserve nor predict hypoxemia during ETI [26]. SpO_2_ values may even be falsely reassuring: in our study, the median SpO_2_ at the end of preoxygenation was 100% [99–100] in both groups. The fraction of oxygen in expired air (FeO_2_) can also provide information on the oxygen reserve [27]: an FeO_2_ of 90% is taken to indicate denitrogenation of the functional residual capacity (FRC). In the operating room, preoxygenation for 3 min is usually sufficient to bring FeO_2_ up to 90%. However, this technique has limitations in the emergency setting, as it is sensitive to leaks, and is not available in the ICU [13]. Moreover, in critically ill patients, particularly those with acute hypoxemic respiratory failure (who were excluded for this proof-of concept study), FeO_2_ may not reliably reflect the effectiveness of preoxygenation: the reduction in functional lung volume leads, on the one hand, to a reduction in FRC and, on the other hand, to shunting that impairs the efficiency of the alveolar–capillary interface [28]. The result is that both SpO_2_ and FeO_2_ can be high despite PaO_2_ being low. PaO_2_ can be considered the reference standard for evaluating the effectiveness of preoxygenation but, unfortunately, cannot currently be obtained at the bedside in real-time in clinical practice [13].

We chose the SpO_2_/FiO_2_ ratio as the inclusion criterion because it was simpler to use than the PaO_2_/FiO_2_ ratio, as obtaining an arterial blood sample may be difficult in emergency situations. The 214 cut-off was chosen as corresponding to a PaO_2_/FiO_2_ ratio greater than 180 [29]. Including patients with more severe hypoxemia may not have allowed for ORI increases to occur in both groups during preoxygenation. We defined mild hypoxemia as SpO_2_ below 97%, as this value corresponds to the inflection point towards a rapid decrease in SpO_2_ during the apneic period [24] and to an ORI value of 0 indicating an absence of oxygen reserves [18].

The clinical implications of our findings may be important. The 81-s forewarning may allow immediate intubation, early face-mask ventilation, insertion of a supraglottic device, or a call for help in the event of intubation difficulties. In addition, ORI monitoring can help identify patients who do not increase their oxygen reserve despite preoxygenation and are therefore at risk of desaturation during ETI. These patients may benefit from a longer preoxygenation period and/or a change in device. An ORI decline might lead to the detection of a fault in the preoxygenation technique such as an insufficient oxygen flow rate or major leaks. The clinical impact of an ORI-guided airway management and preoxygenation strategy remains to be evaluated prospectively.

Our work has several limitations. First, the sample size is small. We had estimated that at least 35 patients had to be included in the analysis to compute the median time from the ORI decrease to the SpO_2_ decrease. However, only 10 of our patients exhibited both ORI  <  0.4 and SpO_2_  <  97% during intubation, and 10 patients did not have an ORI increase  >  0.4 during preoxygenation. Second, 5 patients, representing 9% of the initial cohort, were excluded secondarily due to either a malfunction of the ORI system or a failure of the Rad7^®^ monitor to record the ORI. Third, at the beginning of preoxygenation, the ORI value had already started to increase and was therefore not equal to 0 in either group. However, there was no significant difference in ORI values at this time point between the two groups. Fourth, our results cannot be generalised to all ICU patients. We selected patients with SpO_2_  <  97% and no haemodynamic failure who required ETI mainly to protect the upper airway due to neurological failure. The incidence of hypoxemia during ETI is lower in this population than in patients with respiratory failure. Studies in patients with hypoxemia are therefore necessary. Fifth, as with pulse oximetry, ORI measurement can be unreliable in situations where the peripheral perfusion is impaired, such as in the event of shock or high-dose vasopressor therapy. Sixth, significant inter-individual variability is likely, as some factors may affect the calculation of ORI and therefore alter the relationship between PaO_2_ and ORI. These factors include oxygen consumption, cardiac output, temperature, pH, PaCO_2_, presence of abnormal haemoglobin, and venous pulsatility.

## Conclusion

The median time between the ORI decrease below 0.4 and the SpO_2_ decrease below 97% during the apneic period was 81 s [34–146]. A higher ORI during preoxygenation was independently associated with a lower risk of mild hypoxemia (SpO_2_  <  97%). Whether these findings apply to hypoxemic patients, and the clinical impact of a preoxygenation strategy based on ORI monitoring, remain to be evaluated prospectively.

## Supplementary Information

**Additional file 1: Figure S1.** Study flowchart.

**Additional file 2: Figure S2.** Changes in SpO_2_ and the oxygen reserve index (ORI) in the subset of patients who had moderate hypoxemia (SpO_2_ < 90%).

**Additional file 3: Figure S3.** Changes in SpO_2_ and the oxygen reserve index (ORI) in the overall cohort.

## Data Availability

The study data will be made available upon reasonable request to the corresponding author.
