# Supplementary figures and images for: Oxygen reserve index for non-invasive early hypoxemia detection during endotracheal intubation in intensive care: the prospective observational NESOI study
Source: Ann Intensive Care. 2021 Jul 17;11:112. doi: 10.1186/s13613-021-00903-8 (PMC8374021; doi:10.1186/s13613-021-00903-8)

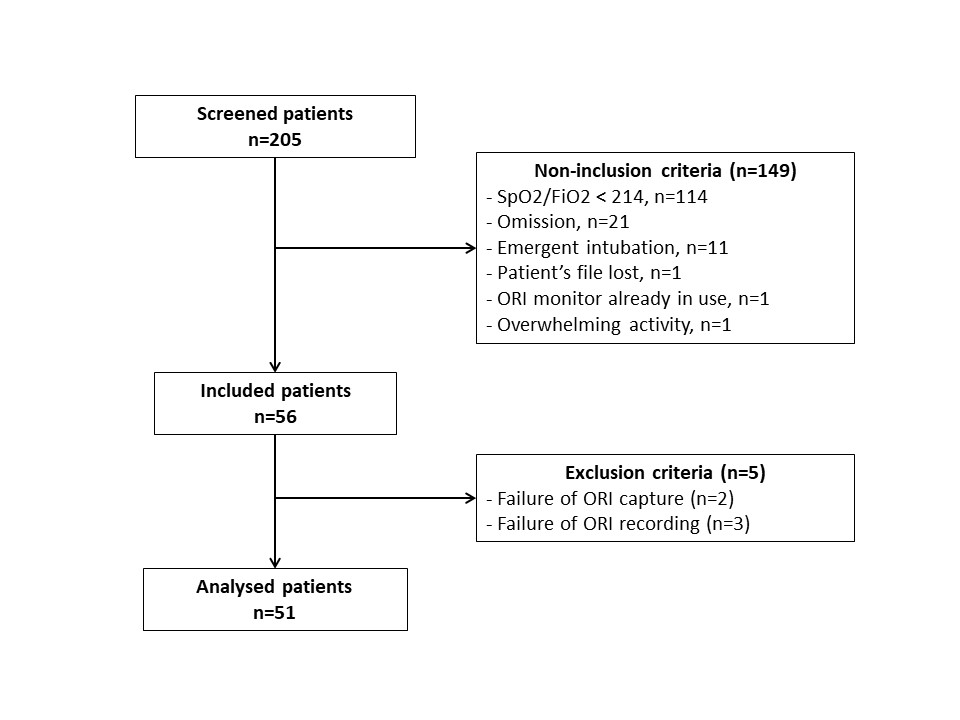

Supplement: Supplementary file 1 — Additional file 1: Figure S1. Study flowchart. [file 13613_2021_903_MOESM1_ESM.jpg]

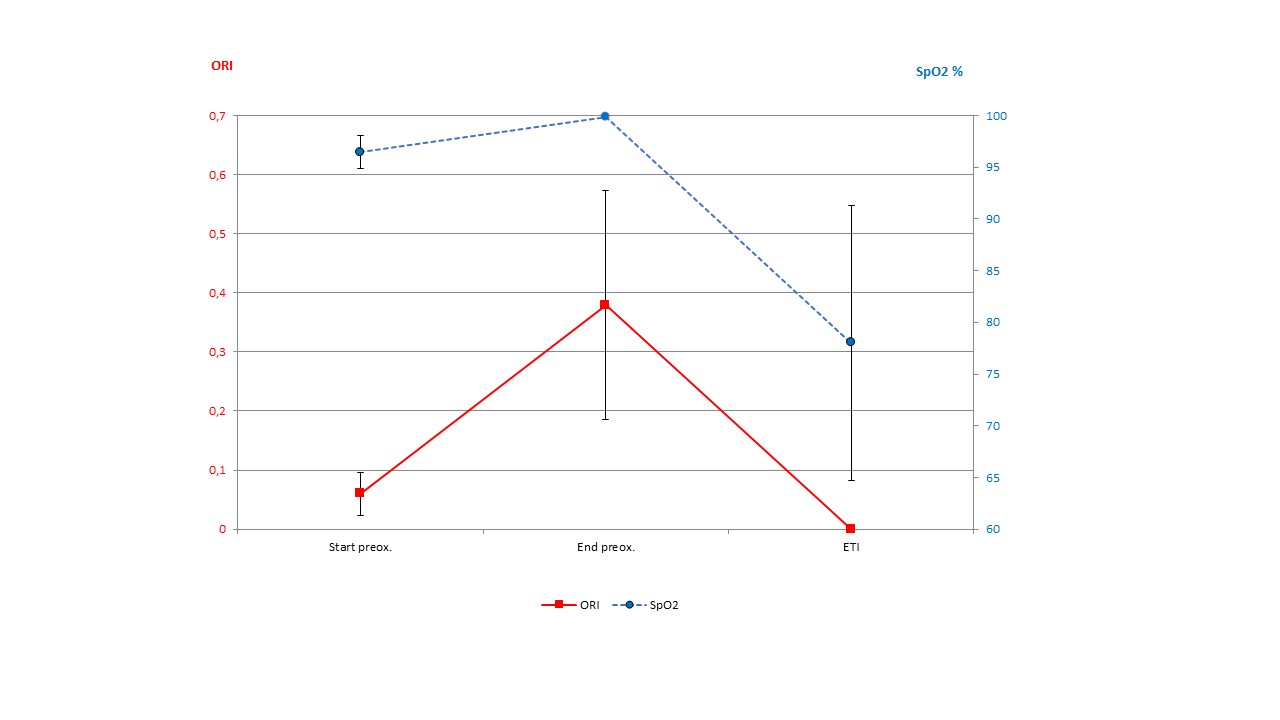

Supplement: Supplementary file 2 — Additional file 2: Figure S2. Changes in SpO2 and the oxygen reserve index (ORI) in the subset of patients who had moderate hypoxemia (SpO2 < 90%). [file 13613_2021_903_MOESM2_ESM.jpg]

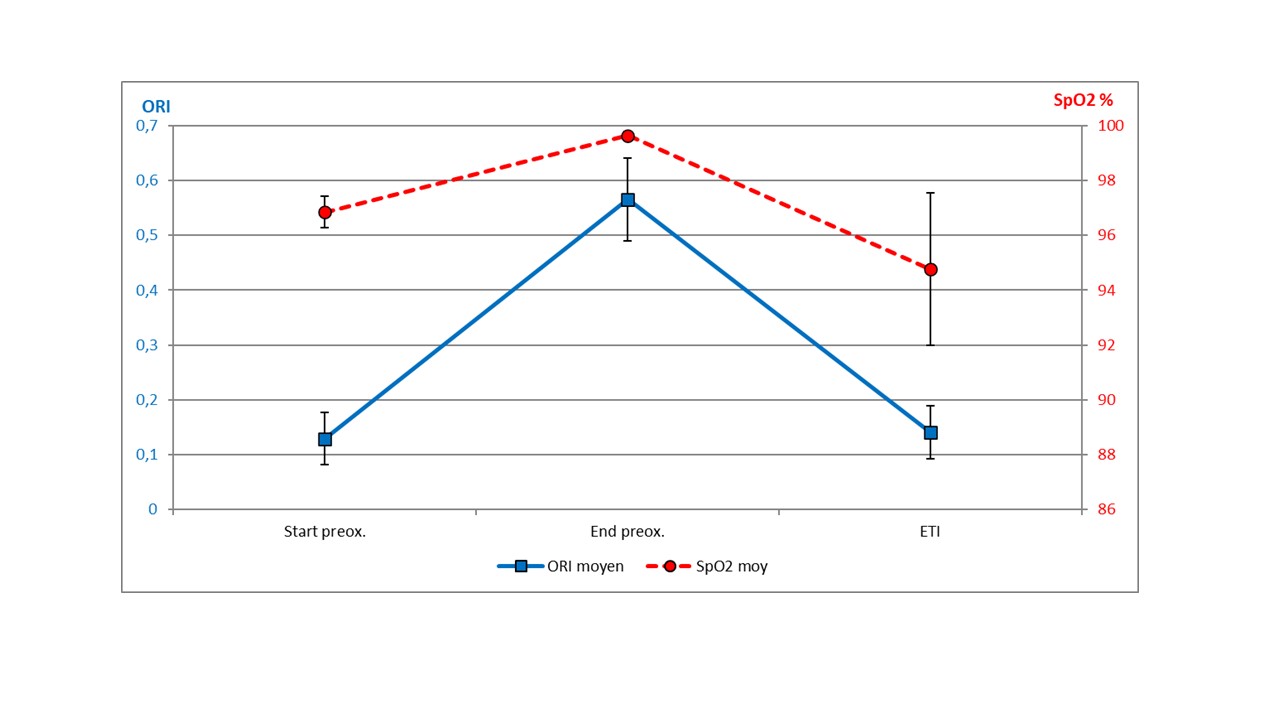

Supplement: Supplementary file 3 — Additional file 3: Figure S3. Changes in SpO2 and the oxygen reserve index (ORI) in the overall cohort. [file 13613_2021_903_MOESM3_ESM.jpg]
